# Zika virus serological diagnosis: commercial tests and monoclonal antibodies as tools

**DOI:** 10.1590/1678-9199-JVATITD-2020-0019

**Published:** 2020-11-18

**Authors:** Isaura Beatriz Borges Silva, Aldacilene Souza da Silva, Mariana Sequetin Cunha, Aline Diniz Cabral, Kelly Cristina Alves de Oliveira, Elizabeth De Gaspari, Carlos Roberto Prudencio

**Affiliations:** 1 Center of Immunology, Adolfo Lutz Institute, São Paulo, SP, Brazil.; 2 Interunits Graduate Program in Biotechnology, University of São Paulo (USP), São Paulo, SP, Brazil.; 3 Division of Vector-Borne Diseases, Adolfo Lutz Institute, São Paulo, SP, Brazil.

**Keywords:** Zika virus, Flaviviruses, Serological diagnosis, Commercial tests, Monoclonal antibody

## Abstract

Zika virus (ZIKV), an emerging arthropod-borne virus (arbovirus) of the *Flaviviridae* family, is a current issue worldwide, particularly because of the congenital and neurological syndromes associated with infection by this virus. As the initial clinical symptoms of all diseases caused by this group are very similar, clinical diagnosis is difficult. Furthermore, laboratory diagnostic efforts have failed to identify specific and accurate tests for each virus of the *Flaviviridae* family due to the cross-reactivity of these viruses in serum samples. This situation has resulted in underreporting of the diseases caused by flaviviruses. However, many companies developed commercial diagnostic tests after the recent ZIKV outbreak. Moreover, health regulatory agencies have approved different commercial tests to extend the monitoring of ZIKV infections. Considering that a specific and sensitive diagnostic method for estimating risk and evaluating ZIKV propagation is still needed, this review aims to provide an update of the main commercially approved serological diagnostics test by the US Food and Drug Administration (FDA) and Brazilian National Health Surveillance Agency (ANVISA). Additionally, we present the technologies used for monoclonal antibody production as a tool for the development of diagnostic tests and applications of these antibodies in detecting ZIKV infections worldwide.

## Background

Zika virus (ZIKV) is an RNA virus of the *Flavivirus* genus, *Flaviviridae* family, that belongs to the arbovirus group, which comprises viruses that share a cycle of transmission via arthropod vectors, most commonly mosquitoes, ticks and flies [1,2]. Other viruses that belong to the *Flavivirus* genus are also some of the most globally relevant viruses in relation to vector-borne diseases, causing worldwide morbidity and mortality, including Dengue virus (DENV) subtypes 1, 2, 3, and 4 and Yellow Fever virus (YFV). ZIKV transmission primarily occurs through the bite of *Aedes* mosquitoes infected with the virus. One of the most alarming features of ZIKV is that it can be transmitted vertically from mother to fetus during pregnancy or at the time of birth, which differs from other flaviviruses [3]. Moreover, ZIKV exhibits long semen persistence, which is associated with its ability to be transmitted sexually, with great potential for propagation.

Most people infected with ZIKV have no symptoms, and when present, the symptoms are mild, such as headache, skin rash, fever, joint pain, muscle ache, retro-orbital pain and conjunctivitis [4]. It is important to emphasize that these symptoms are non-specific and common to several other infectious diseases, including other arboviruses, such as DENV, YFV, Chikungunya virus (CHIKV), among others. Since infected individuals generally do not present severe illness, they therefore do not seek medical assistance, though death due to ZIKV is rare [5,6]. For this reason, many people may not realize that they have been infected.

ZIKV was isolated for the first time in 1947 in the Ziika forest near Lake Victoria in Uganda [7]. The first documented outbreak of ZIKV outside of the African continent was described in 2007 in Micronesia. However, there were no reports of severe cases described in the literature [8,9]. In late 2013, an increase in the incidence of Guillain-Barré syndrome was observed in French Polynesia after a high number of ZIKV infections were identified during the same period [10].

Subsequently, a small outbreak in the Northeastern Brazil was described in 2015 [11], and in October of the same year, an unusual increase in microcephaly cases in newborns was observed in Brazil, especially in the northeastern region [12]. In response, the country declared a national public health emergency in November [10,13]. On February 1, 2016, the World Health Organization (WHO) declared that such complications associated with ZIKV infections constitute a Public Health Emergency of International Concern [14].

ZIKV can be grouped into two main strains: African and Asian. Phylogenetic analyzis indicate that ZIKV originated in Africa and then spread to Asia, the Pacific islands and throughout the Americas. The introduction of ZIKV in the Americas was probably due to a single introduction of an Asian strain between May and December 2013, more than 12 months before the detection of ZIKV in Brazil [15]. Some studies have already demonstrated intrinsic differences in pathogenicity/virulence between the African and Asian lineages. The Asian strain has a lower infection rate, lower viral production and low cell death induction that may contribute, at least in part, to the ability to cause persistent infections in the central nervous system of fetuses [16-17].

The structure of ZIKV is very similar to that of other flaviviruses. The structural proteins include the envelope protein (E), capsid protein (C), membrane precursor (prM) and membrane protein (M). ZIKV protein E is the main viral protein involved in cell receptor binding and entry and, therefore, is considered to be one of the major determinants of ZIKV pathogenesis [18]. Each monomer of protein E contains three ectodomains: domains I, II and III (DI, DII and DIII). These domains are involved in such functions as cell receptor virus binding and fusogenic properties and play a critical role in neutralizing antibody stimulation [19].

The nonstructural protein 1 (NS1) protein is also considered an important antigenic marker of ZIKV and other flaviviruses. NS1 is a glycoprotein that exists as a membrane-associated dimer after translocation to the lumen of the endoplasmic reticulum of virus-infected cells. As the genetic material and viral replication complex are also located in the endoplasmic reticulum, this host cell organelle is essential for flavivirus RNA replication [20]. In addition, infected cells secrete NS1 as a hexameric lipoprotein that interacts with complement system proteins and has many immune system modulation functions that contribute to evasion [20].

The incidence of ZIKV in the Americas peaked in 2016 and decreased substantially over the course of 2017 and 2018, with a slight increase in 2019 [21]. ZIKV transmission has been identified in all North and South American countries, except for Canada. It is noteworthy that in Brazil, 17,041 suspected cases of growth and developmental changes in fetuses that were possibly related to ZIKV infections and other infectious etiologies were reported between 2015 and 2018, with 2865 confirmed cases [22]. Since 2015 until epidemiological week 53 of 2019, the number of cumulative cases across the Americas was 857,648 [23]. In Brazil, 10,768 probable cases were reported throughout 2019 [24]. 

Epidemiological data contribute to assessing the incidence of infections and their context and complexity, assisting in the setting of goals and selection of necessary interventions [25]. Nonetheless, obtaining reliable epidemiological data is directly related to the efficiency of adequate diagnosis of infections. Concerning flavivirus infections, there is a limitation due to its co-circulation in certain areas, high similarity in clinical symptoms and cross-reactivity in laboratory diagnostic methods. Studies using the Notification Disease Information System (SINAN) database during the period from 2015 to 2017 showed that an increase in individuals reported having ZIKV infections may have contributed to a rise in misreported DENV cases, indicating a scenario in which people infected with Zika were erroneously classified as having DENV infection and vice versa [26].

Overall, inadequate diagnosis can interfere with the risk estimation, propagation, and determination of the true impact of ZIKV infection on other arboviruses and, consequently, on an efficient response from public health agencies. Accordingly, this review aims to provide an update of the main commercial serological diagnostic test approved by the US Food and Drug Administration (FDA) and Brazilian National Health Surveillance Agency (ANVISA). This review also aims to present the advantages of monoclonal antibodies as tools for diagnosis, their recent applications in the detection of ZIKV infections and other perspectives regarding ZIKV diagnosis.

## Zika Virus Diagnostics

The most appropriate diagnostic test for the detection of viral infections is dependent on the stage of the disease, which is divided into acute and convalescent phases. The acute phase is characterized by the early stages of infection when viruses replicate in infected cells and the host develops viremia. After the onset of clinical manifestations, there is an initial response to the infection by the production of IgM antibodies against the virus; this immune response is also considered part of the acute phase. The convalescent phase occurs in the late stages of infection, and a more specific and persistent IgG antibody response against the virus develops [27,28]. The ideal diagnostic test should have high sensitivity and specificity, which is the ability of the test to correctly identify an individual with the disease and the ability to correctly classify an individual without the disease, respectively. These terms are also defined by the equations: sensitivity = true positives/(true positives + false negatives) and specificity = true negatives/(true negatives + false positives). However, in general, these measures are inversely proportional, meaning that the higher the sensitivity, the lower the specificity, and vice versa [29]. Diagnostic tests can consist of molecular or serological assays. The former are direct assays used for detection and/or quantification of genetic variants, i.e., they are based on the presence of viral nucleic acids in bodily fluids [30]. In contrast, serological assays can be employed to indirectly identify the previous circulating virus and to measure the patient's immune response against the virus by detecting antibodies against the virus in serum. Demonstration of the causative organism or a specific antibody is required for diagnosis of any infection [31]. ZIKV RNA may be detectable in serum for approximately 4-7 days following the onset of symptoms. However, it has been demonstrated that ZIKV RNA remains detectable in serum for approximately 54 days after symptom onset, in urine for 39 days, and in semen for 120 days [32]. Indeed, semen appears to be the fluid in which the virus persists the longest. Nicastri et al. [33] and Barzon et al. [34] reported the detection of viral RNA in semen 188 and 370 days after symptom onset, respectively.

Although IgM levels vary, they are generally positive from the fourth day after the onset of symptoms until up to 12 weeks, and the levels may persist for even longer. The IgG response develops shortly after the IgM response, and it has been shown that IgG levels remain high for at least 2 years after infection [35]. When patients have symptoms and visit clinics, viremia is often already low or undetectable, imposing a narrow diagnostic window for the detection of viral components [36]. Thus, serological diagnosis via antibody detection is an efficient approach to determining infection status over long periods. Serological assays are able to detect ZIKV infection in cases in which virus nucleic acids are no longer detectable. This is partly due to the period in which patients seek medical attention after the onset of symptoms or return from traveling to a ZIKV-affected country [37]. Despite evidence of prolonged persistence of ZIKV nucleic acids in body fluids [38], this genetic material is generally not consistently detectable in serum and urine for prolonged periods. 

Zika virus shares approximately 55.6% amino acid sequence identity with DENV, 46.0% with YFV, 56.1% with Japanese Encephalitis virus (JEV) and 57.0% with West Nile virus (WNV) [39]. This large similarity between flaviviruses often display antibody cross-reactivity, as they share multiple conserved epitopes that can act as a key target for cross-reactive human antibody responses [40]. Considering the co-circulation of flaviviruses in certain geographic areas, the pre-existence of antibodies against some flaviviruses represents a great challenge for understanding the immune response to and pathogenesis of the viruses. Thus, detection tests for IgG against ZIKV are not reliable due to the potential cross-reactivity in those with previous infections by other flaviviruses [37], hindering the ability to fully assess a patient's serological profile. Studies made using Tick-Borne Encephalitis (TBE) and/or YFV vaccinated individuals’ samples evaluated the effect on the patterns of antibody responses in primary ZIKV infections. The results showed that pre-existing cross-reactive immunities had a strong influence on the antibody responses in primary ZIKV infections, resulting in higher titers of broadly flavivirus cross-reactive antibodies and alteration in ZIKV-specific antibodies levels [41]. 

Although ZIKV and DENV show approximately 41% to 46% differences in envelope protein amino acid sequence [42], the similarities are sufficient to allow cross-reaction between ZIKV and DENV, and a number of reports demonstrate the difficulty in distinguishing DENV and ZIKV infections serologically [43-46]. Furthermore, the cross-reactivity of flavivirus antibodies associated with co-circulation represents a great challenge in obtaining specific and sensitive diagnostic tests for each virus of the *Flaviviridae* family. In addition, similar clinical manifestations, and even the presence of many asymptomatic patients, make it even more difficult to accurately diagnose ZIKV.

Public health surveillance monitors infectious diseases in the population. Thus, diagnostic tests has a strong role in providing accurate results that allow pathogen occurrence identification so that measures can be executed to control and prevent them from reappearing. Particularly, serological test is a way to better understand the expansion of the infection through the population, allowing a serosurveillance on a herd level. Tests should be easy to use and provide a rapid result to have a positive impact on care [47]. The dynamics of testing infectious diseases needs to act as a bridge between the laboratory and public health organs to support surveillance activities. Surveillance case data applied to perform epidemiological mapping, using geographical information system (GIS) approach, can be helpful for a preventive and control strategies [48].

## Approved and Commercial Serologic Tests for Zika Virus

Given the necessity of establishing strategies for the control and dispersion of ZIKV, the Centre for Disease Control and Prevention (CDC) has established recommended guidelines for ZIKV diagnosis. The diagnostic tools consist of reverse transcriptase reaction assays followed by real-time polymerase chain reaction (RT-qPCR), ZIKV IgM antibody capture immunoenzyme assays (MAC-ELISAs) and plate reduction neutralization tests (PRNTs) [8,49].

The RT-PCR assay is only applicable during the acute phase of infection, when viral RNA is still detectable in body fluids, and the persistence of viral RNA varies according to the biological material examined. The sensitivity of the RT-PCR assay is very important to avoid false negative results [50]. Up to ten mismatches have already been identified between the nucleotide sequences reported in published assays and the consensus sequence of the Asian ZIKV strain, in addition to mismatches in primers and probes used in the RT-PCR amplification. Such inconsistencies are a potential limiting factor for the sensitivity of the test due to the existing genetic variability in the Asian strain [50,51]. Therefore, there must be a continuous surveillance to detect new ZIKV variants and an update in molecular methods by modifying the primer and probe sequences to overcome the impact of the mismatches mutations and improve the detection sensitivity.

MAC-ELISA is a serologic test used for qualitative detection of IgM antibodies in the serum or cerebrospinal fluid. Nonetheless, the results can be difficult to interpret due to the possible non-specific reactivity of antibodies. Consequently, tests determined to be positive, equivocal or inconclusive should be confirmed by PRNT, a serological test based on the ability of specific antibodies present in the serum of patients to neutralize viruses by preventing plaque formation in a cell monolayer. PRNT is currently considered the "gold standard" for differential flavivirus serodiagnosis due to its high specificity. However, this assay has a high cost, requires highly specialized laboratories with adequate equipment to maintain cell culture, and special regulations for working with the active virus; it is also difficult to perform, and 5 to 10 days are required to obtain results [49,52].

Among the available serological commercial tests, the tests developed by Euroimmun AG (Germany) and InBios (USA) are noteworthy. The Euroimmun assay was the first commercially available serological test for ZIKV detection, and it has been extensively evaluated in the literature [53-56]. The anti-Zika virus IgM/IgG/IgA ELISA is based on an ELISA using the Zika virus NS1 protein for the detection of IgM, IgG and IgA in serum samples. Studies such as those by Huzly et al. [53] reported high specificity of this test using different serum samples from patients with previous flavivirus infections. Additionally, L'Huillier et al. [54] conducted a comparative study between Euroimmun IgM and IgG ELISAs and MAC-ELISA and subsequent PRNT for the confirmation of positive or inconclusive results. It was demonstrated that Euroimmun's combined IgG/IgM test presented good specificity (95%) that was even better than that of MAC-ELISA, though the sensitivity of this test was significantly lower than that of MAC-ELISA (39.5%).

The InBios assay, also known as the ZIKV Detect 2.0 The IgM Capture ELISA kit, is an assay based on capture ELISA for qualitative detection of IgM antibodies against ZIKV using the viral envelope protein as an antigen. This assay was the first commercial serological test to receive FDA marketing authorization in the USA, granted in May 2019 [57]. Granger et al. [58] and Safronetz et al. [59] demonstrated that the InBios test provides diagnostic results comparable to those of the CDC MAC-ELISA and still exhibited high sensitivity (100%). The low sensitivity observed with the Euroimmun assay may be due to the high specificity of ZIKV antibodies. Specificity is a critical factor for a diagnostic test, as sensitivity is an important feature in determining its usefulness, and low sensitivity can lead to false negative results. Although additional studies with a larger panel of samples are still needed, these tests have great potential for the serological evaluation of ZIKV infections with reduced time for confirmation of infection, and these tests may decrease the need for PRNT confirmation tests.

The ADVIA Centaur Zika test was the second Zika diagnostic test that the FDA has allowed to be marketed in the USA for detecting ZIKV IgM antibodies. The third and last authorized test to be marketed was LIAISON XL Zika Capture IgM Assay II. Previously, these tests had only been authorized for emergency use under the FDA’s Emergency Use Authorization (EUA) authority. A unique serological test that is still under the FDA’s EUA is DPP Zika IgM Assay System from Chembio Diagnostic Systems [60]. In Brazil, ANVISA has also approved the registration of some of these commercial tests to broaden access to diagnosis and allow for greater monitoring of ZIKV infection. Currently, there are 48 tests approved by ANVISA; 36 are serologic tests, some of which are described in Table 1 [61]. Most of them present elevated sensitivity and specificity. However, these tests are generally not validated using samples from more than two different countries or regions, limiting their use in a wide and universal way due to the different circulating strains of ZIKV. Different ZIKV isolates may present genotypic and phenotypic variations that influence the manner by which the immune system responds and thus the antibody response to the virus [62]. Overall, these assays have good prospects for use in routine diagnostic laboratories if they pass for a systematic clinical evaluation.

**Table 1.** Main currently approved ANVISA tests.

Test	Company	Test format	ZIKV antigen	Sensitivity	Specificity
DPP Zika IgM/IgG Assay System	Chembio Diagnostic Systems (USA)	Immunochromatographic	NS1	IgM: 89.5% IgG: 97.5%	IgM:97.7% IgG: 98.3%
RecombiLISA Zika IgM ELISA Kit	CTK Biotech (USA)	ELISA	NS1	94.7%	98.5%
ZIKV IgM ELISA kit	DIA.PRO Diagnostic Bioprobes Srl (Italy)	ELISA	-	IgM: 69% IgG: 80.5	IgM: 96% IgM: 94%
LIAISON XL Zika Capture IgM Assay	DiaSorin (Italy)	Microparticle antibody capture chemiluminescence immunoassay	NS1	100%	91.2%
Zika ELISA IgM/ IgG	Vircell S. L. (Spain)	ELISA	-	IgM and IgG: 91%	IgM and IgG: 99%
ZIKV-DENV-CHIKV IgM/IgG IFA		Immunofluorescence	Infected cells	IgM: 93.3% IgG: 94.7	IgM: 94.4% IgG: 94%
Anti-Zika virus ELISA IgM/IgG	Euroimmun (Germany)	ELISA	NS1	IgM: 87% IgG: 100%	IgM and IgG: 97%
IIFT Arboviral Fever Mosaic 2 IgM/IgG		Immunofluorescence	Infected cells	IgM: 96.9% IgG: 96.8%	IgM: 98.1% IgG: 93.4%
NovaLisa Zika Virus IgM µ-capture	NovaTec Immunodiagnostica GmbH (Germany)	ELISA	NS1	98.5%	100%
Elecsys® Zika IgG	Roche Diagnostics (Switzerland)	ELISA	-	93.11%	99.82%
STANDARD E Zika IgM	SD Biosensor Inc. (South Korea)	ELISA	-	100%	-
STANDARD Q Zika IgM/IgG		Immunochromatography	-	IgM: 98% IgG: 75.9%	IgM: 100% IgG: 70%
Zika IgG/IgM	Ebram Laboratory Products (Brazil)	Immunochromatography	-	99.9%	98.9%
Imuno-Rapid Zika IgG/IgM	Wama Laboratory Products (Brazil)	Immunochromatography	ZIKV inactivated	96.2%	IgG: 99.1% IgM: 98.2%
Allserum Zika IgM	Mbiolog Diagnostic (Brazil)	ELISA	NS1	100%	94.4%
Kit Xgen Zika Virus IgG/IgM	Mobius Life Science Industry and Commerce of Laboratory Products (Brazil)	ELISA	NS1	IgG: 100% IgM: 98%	IgG: 98% IgM: 98%
OL Zika Ag NS1	Orangelife Commerce and Industry (Brazil)	Immunochromatography	-	90.2%	99.5%
OL Zika IgM/IgG		Immunochromatography	-	IgM: 93% IgG: 94%	IgM: 97% IgG: 98%
Zika IgG/IgM Rapid Test	Diagnostic Industry and Commerce (Brazil)	Immunochromatography	-	-	-
ECO F Zika IgG/IgM	Eco Diagnostic Ltda (Brazil)	Immunofluorescence	-	98%	99%
ECO F Zika Ag		Immunofluorescence	NS1	97%	97%
ZiKa IgG/IgM ECO Teste		Immunochromatography	-	97.38%	IgM: 100% e IgG: 96.34%

NS1: Nonstructural protein 1.

## Monoclonal Antibodies Used in Zika Virus Diagnosis

Monoclonal antibodies (MAbs) are products of individual B-cell clones. They have broad applicability in therapies and drug targeting, and have a profound impact on the immunodiagnostics of infections. MAbs interact with a single antigenic determinant, allowing for specific reactivity and accurate identification of the organism of interest. This feature confers a great advantage to MAbs versus polyclonal antibodies, which have different epitope specificities and affinities [63,64]. Moreover, MAbs are relatively easy to use and introduce into trials.

Regarding MAb production, advances in molecular biology and genetic engineering over the years have led to different methods of producing and modifying these antibodies, as opposed to the traditional technique of hybridoma production. Some of these MAbs are produced through cell-free libraries, combinatorial synthetic libraries, affinity maturation, and production in transgenic animals and plants, as well as several other technologies, allowing for great robustness and interaction efficiency with a specific target [64]. 

The development of MAbs has been mainly focused on the development of therapies for cancer, autoimmune diseases, and inflammatory conditions. However, the application of MAbs for infectious diseases still has limitations [65]. One of the main reasons for this scenario is the economic viability for MAb production against targets that may cause an episodic disease, hindering their continued production. MAb production generally involves stages of establishment and optimization of cell culture process, following antibody production, purification, and polishing steps. The costs of the final product can still be a limiting factor for commercial manufacturing. Meanwhile, alternatives have been made to maximize product yield and to improve the robustness [66], in order to enable MAbs application expansion in the field of infectious diseases.

**Figure 1.**
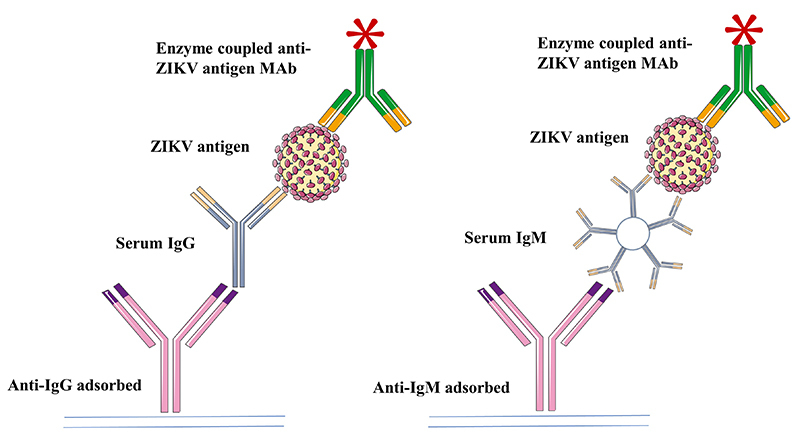
Schematic representation of an antibody capturing serology method for detection of IgG and IgM antibodies in serum sample using a monoclonal antibody that recognizes ZIKV antigen.

Figure 1 illustrates an antibody-capture assay, representing how monoclonal antibodies can be used for a serology assay to detect IgM or IgG antibodies in patient’s serum samples. The capturing method typically employs a capture antibody, anti-IgG or anti-IgM, coated in a surface, then serum sample is incubated, followed by addition of ZIKV antigen and a specific anti-ZIKV MAb conjugated with an enzyme. A substrate for the enzyme is then added and, after a short incubation, the signal is measured.

Despite the worldwide effort to facilitate flavivirus diagnostics, no fully validated serological test shows good sensitivity and specificity without the interference of false positive or negative results and is also robust and rapid. Accordingly, the search for MAbs that specifically recognize each virus of this family is extremely relevant and has been addressed by many researchers. Table 2 presents the main monoclonal antibodies against ZIKV currently reported in the literature. To date, there are 21 MAbs that mainly target ZIKV E protein, with DIII being the predominant epitope. All these MAbs demonstrate neutralizing activity or specific detection. Table 3 lists selected studies in which some of these MAbs have been applied in diagnostic tests for ZIKV.

**Table 2.** Main monoclonal antibodies against ZIKV.

Source	MAb	Epitopes
Murine	1 (2A10G6) [67]	*Fusion Loop*
Human	1 (ZKA64) [68]	DIII
Murine	2 (ZK54/ZV67) [69]	DIII/DIII
Human	3 (Z3L1/Z23/Z20) [70]	DI, DII/ DIII, DI/DIII
Human	1 (ZIKV-117) [71]	DII
Murine	1 (ZV-2) [72]	E
Human	1 (Z004) [73]	DIII
Human	1 (ZKA35) [74]	NS1
Human	2 (m301/m302) [75]	DIII
Murine	1 (1F12) [76]	NS1
Murine	2 (J5E1/J2G7) [77]	E/NS1
Human	1 (ZK2B10) [78]	DIII
Human	3 (P1F12/P1H09/P1804) [79]	E
Human	1 (ZIKV-195) [80]	DI/DII

MAb: monoclonal antibody; E: envelope protein; DI: envelope domain I; DII: envelope domain II; DIII: envelope domain III; NS1: nonstructural protein 1.

**Table 3.** Monoclonal antibodies used in ZIKV diagnostic tests.

MAb	Molecule(s) detected	Test format	Method of MAb isolation	Sensitivity	Specificity
ZV-2 [72]	E protein	Electrochemiluminescence	Hybridoma	1 PFU in 100 μl of samples	-
Anti-ZIKV NS1 [81]	NS1 protein	Immunochromatography	Hybridoma	81%	86%
ZKA35 [74]	NS1 protein	Blockade-of-binding ELISA	Memory B cells from infected individuals	95%	91.9%
1F12 [76]	NS1 protein	Double-antibody sandwich ELISA	Hybridoma	99.8%	-
J5E1 and J2G7 [77]	IgG and IgM	Immunochromatography	Hybridoma	99% IgG 96.7% IgM	99.3% IgG 98.7% IgM
P1F12, P1H09 and P1804 [79]	Zika particles	ELISA	Plasmablast from infected individuals	-	-

MAb: monoclonal antibody; E: envelope protein; NS1: nonstructural protein 1; PFU: plaque-forming unit.

Among the tests referred in Table 3, the rapid test developed by Kim et al. [77] was the first rapid test to be developed and the first test to receive approval from ANVISA with cooperation of BahiaFarma (Bahia, Brazil) and GenBody Inc. (Cheonan, Korea). Regarding MAb obtention techniques, hybridoma production is the most well established, is considered the most traditional methodology and is still the most widely used. This technique is based on the fusion of B lymphocytes with myeloma cells to generate hybrid cells that continuously produce antibodies in vitro [82]. However, the steps for producing hybrid cells are laborious and dependent on immune response induction. Alternatively, techniques involving isolation of infected plasmoblasts or memory B cells from infected individuals have been widely employed. The great advantage of these methodologies is the isolation of antibodies from donors who carry antibodies derived from cells that were activated naturally, allowing for full exploration of the strength of the human antibody response to a pathogen [83].

The MAb developed by Balmaseda et al. [74] is derived from a panel of MAbs produced by immortalization of memory B cells using Epstein-Barr virus from four infected ZIKV donors of the recent epidemic. Robbiani et al. [73] and Sapparapu et al. [71] also isolated MAbs by expanding memory B cell clones from ZIKV-infected individuals. Prior characterization of these clones was performed based on their ability to bind viral proteins, such as NS1 and E, and their ability to neutralize ZIKV infection.

In addition to the methodologies for MAb obtention, phage display has emerged as one of the main alternatives for the generation of human recombinant MAbs. Phage display enables to select human MAbs without *in vivo* immunization through the selection of combinatorial libraries of human antibodies displayed on filamentous phage surfaces against a target antigen, allowing for rapid and economical MAbs generation [84]. The phage display biopanning process mimics B cell clonal selection of the immune system by enriching phage particles that express antibodies with a desired specificity [85]. Therefore, the technique is highly robust due to the high stability of the phages, allows for the control of biochemical parameters throughout the selection process, and can shape the specificity profile of an antibody from the beginning. Wu et al. [75] identified a panel of human MAbs with high affinity and specificity for ZIKV DIII from a phage display naïve antibody library.

Phage display is also considered an important tool for mapping the epitopes of monoclonal antibodies. In this regard, Ravichandran et al. [85] explored different approaches using whole-genome fragment phage display libraries covering the entire ZIKV genome. From this library, the authors mapped some ZIKV-specific MAbs, selected ZIKV-E-specific antibodies from the serum and urine of infected patients and performed comprehensive antibody repertoire analyses of these samples, allowing for the assessment of the immune response against viral infections and the identification of targets for serodiagnosis.

The abovementioned techniques include different antibody formats, such as whole antibodies, fragment antigen binding (Fab) or single-chain variable fragments (scFv). Each of these formats has advantages and disadvantages based on the desired application. For diagnostic methods, such as immunohistochemistry, the lack of Fc ensures the reduction of non-specific binding in addition to a good tissue distribution [86]. Given this advantage, single-domain antibodies (sdAbs) have emerged with great potential for diagnostic applications, mainly due to their high stability and ability to recognize cavities and cracks in the surface of proteins that cannot be recognized by conventional recombinant antibody formats. In addition, these antibodies have a low cost and are relatively easy to produce compared with other antibody formats [87]. Considering the need to develop rapid and effective diagnostic methods and the increasing use of antibody-based health products, sdAbs can be considered an important biotechnological tool for application in the diagnosis of infections with the ability to cause sudden outbreaks, as in ZIKV infection.

## Other Perspectives in the Development of Serological Diagnoses for Zika Virus

Other innovative methodologies have been applied for the development of serological diagnostics with the potential to outperform conventional methodologies in terms of speed and sensitivity. The reporter virus neutralization test (RVNT) represents a very promising alternative to the PRNT. RVNT uses luciferase-labelled ZIKV and DENV, and neutralizing antibodies can be quantified within 24 h instead of the typical 7-day period required for plaques to be visible with the PRNT method [57]. Wang et al. [88] developed a capacitive biosensor using microwires coated with the ZIKV envelope protein for the detection of serum antibodies; this biosensor represents a robust label-free assay that enables rapid diagnosis of infection at the point of care (POC). Mishra et al. [89] used a designed platform of peptide array to identify discriminant epitopes for serodiagnosis of ZIKV infection. Based on results obtained with peptide array, they developed a ZIK NS2B peptide ELISA that presented high sensitivity (96%) and specificity and (95.9%). 

In addition, Kareinen et al. [90] developed a time-resolved Förster resonance energy transfer (TR-FRET) assay involving two chromophore-labelled proteins (ZIKV NS1 protein and a superantigen) that bind simultaneously to an antibody present in a patient's serum. This technique showed high sensitivity and specificity, with the potential to be applied in POC diagnoses. Zhang et al. [91] also constructed a highly multiplexed and programmable peptide array platform containing the ZIKV NS1 and DENV2 antigens on a nanostructured plas monic gold (pGOLD) platform. The chip can capture IgG and IgA antibodies against ZIKV and DENV antigens in patient serum. The pGOLD platform is capable of amplifying near-infrared fluorescence by up to ~ 100 times, allowing for the sensitive analysis of multiple analytes.

## Final Considerations

ZIKV infections constitute a major public health problem in Brazil and around the world, mainly due to the magnitude of its complications, and there are still major challenges in our understanding of ZIKV infection mechanisms. Among them, the lack of complete understanding regarding the risk of complications according to different strains of the ZIKV, possible environmental, genetic or other cofactors that may increase the risk of complications and the lack of knowledge of the role of asymptomatic infections and other modes of transmission play in the general dynamics of circulation. This scenario makes it difficult to fully characterize the damage that ZIKV infections can cause. 

Despite the great advances in serological assays in the last years, the incomplete knowledge about the pre-existing immunity for other flavivirus of the population in endemic countries, may impose difficulties in diagnosis [92]. Some improvements allowing multiplexing of detection assays to numerous arbovirus, providing a serological panel of an individual and high throughput testing, would increase the quality of serologic data generated. Moreover, the implementation of tests that present portable, rechargeable devices and the possibility to be conducted without extensive technical skills in the communities reality, may also facilitate determination of infection spread and the level of care [93]. 

Since the beginning of the ZIKV epidemic, many efforts from health care organizations around the world have been applied to the development of plans for ZIKV control. In 2016, the WHO implemented the Zika Strategic Response Plan, which involves four main objectives to support governments in preventing and managing the complications caused by this virus and mitigating the socioeconomic consequences, including detection, prevention, care and support, and research [94]. In Brazil, different strategies were developed by the Ministry of Health, including developing a National Microcephaly Coping Plan through the mobilization and control of *Aedes aegytpi*, updating surveillance protocols and responding to ZIKV infections and its resulting complications. Moreover, for the first time, the Ministry of Health has organized a network of integration between managers, researchers and civil society to cope with the disease: Renezika. The creation of this network has demonstrated the ability of the Brazilian scientific community to respond to major international health problems and to propose relevant activities such that future emergencies can be prevented with rapid and effective action.

## Conclusion

Despite the limitations in flavivirus serological assays due to high cross-reactivity, many advancements have been made in ZIKV diagnosis, even with the decrease in the number of infected patients. Investment in the development of innovative methodologies to obtain immunobiological products quickly and effectively represents a crucial factor for the advancement of public health systems worldwide. Contemporary molecular biology and molecular immunology technologies, such as antibody engineering and phage display, allow for the possibility of producing a specific human antibody with relatively high affinity to a target molecule in vitro without in vivo immunization. The application of these biomolecules in innovative technologies, such as biosensor chips, with potential application in POC diagnosis may enable increased epidemiological control efficiency. These tests may ensure accurate evaluation of ZIKV infection rates, contributing to the development of efficient public policies to combat this infection.
